# Supplementary material for: Biomarkers in Fabry Disease. Implications for Clinical Diagnosis and Follow-up
Source: J Clin Med. 2021 Apr 13;10(8):1664. doi: 10.3390/jcm10081664 (PMC8068937; doi:10.3390/jcm10081664)
Supplement: Supplementary file 1 [file jcm-10-01664-s001.pdf]

Supplementary Materials

Table S1. Accumulation biomarkers of Fabry disease.

Accumulation biomarkers	Specimen	Value	Availability	Changes or association with response to therapy	Limitations
Gb3 [67, 69, 70, 72]	plasma, urine	Assess diagnosis. Biomarker of burden or global activity of disease	use in clinical practice	Levels decrease with ERT, mainly in males	Limited sensitivity in late-onset forms and/or female. Limited utility as a biomarker of response
Gb3 (CD77) [97, 98, 99]	PBMC [96, 97, 98]. Human tubular cell culture [96]	Good detection capacity in classic forms. In human renal tubular cells, its expression increases by silencing the expression of α-gal A	in vitro, clinical work-up	Levels decrease with ERT	More studies are required to validate its clinical utility
Lyso-Gb3 [11, 12, 13, 66, 71, 73-75,77- 79, 82, 83, 93]	plasma	Assess diagnosis. Biomarker of burden or global activity of disease [67, 71]. Good diagnostic marker for classic phenotype [71, 74, 75, 78]	use in clinical practice	Levels decrease with ERT, mainly in males	May has limited sensitivity in female. Controversy in results obtained in studies of association with clinical manifestations, progression or response
Lyso-Gb3 [67, 76, 89, 91]	urine	Assess diagnosis. Biomarker of burden or global activity of disease [67, 76] correlate with proteinuria and albuminuria. No correlation with GFR [76]	clinical work-up	No marked differences in levels between treated and untreated with ERT	Limited sensitivity in late-onset forms and/or female patients. No correlation with GFR
Lyso-Gb3 analogues [88, 90]	plasma	Assess diagnosis, mainly in males. Biomarker of burden or global activity of disease	clinical work-up	Levels of some analogues are increased in untreated respect treated males with ERT	Do not add clinical value over what of plasma Lyso-Gb3 already has, since the relative ratio of analogs relative to Lyso-Gb3 is lower than that observed in urine
Lyso-Gb3 analogues [89-93]	urine	Assess diagnosis. In males and females, Lyso-Gb3 plus its analogues have diagnostic utility for both classic and non-classic forms. Potential indicators of severity and progression of cardiac injury: Lyso-Gb3 analog (+50) associated with cardiac phenotype [90]. Lyso-Gb3 analogues (+16, +34, +50) associated with LVMI and the MSSI [93]	clinical work-up	Levels of Lyso-Gb3 analogues decrease with ERT. No marked differences in levels between untreated and treated with ERT in females compared to males	Lyso-Gb3 plus its analogues profile has a high interindividual variability. More studies are required to validate its clinical utility
CDH isoforms (Gb2 plus LacCer isomers) [94, 95]	urine	Greater detection capacity in asymptomatic females compared to plasma Lyso-Gb3	clinical work-up	Not studied	More studies are required to validate its clinical utility
Gb2 isoforms [95]	urine	Greater diagnostic sensitivity in females compared to that obtained with the sum of the two isomers	clinical work-up	No differences in levels between untreated and treated females	Methodology used to separate isomers is hardly applicable to clinical laboratory routine. More studies are required to validate its clinical utility
Methylated Gb3 isoforms [96]	urine	Good detection capacity for both classic and no classic forms, including females	clinical work-up	Increased levels in treated respect untreated males with ERT	More studies are required to validate its clinical utility

a-gal A: alpha-galactosidase A; CDH: ceramide dihexosides; ERT: Enzyme replacement therapy; GFR: Glomerular Filtration Rate; Gb2: Galabiosylceramide; Gb3: Globotriaosylceramide; LacCer: Lactosylceramide; LVMI: Left Ventricular Mass Index; Lyso-Gb3: Globotriaosylsphingosine; MSSI: Mainz Severity Score Index.

Table S2. Response biomarkers of Fabry disease.

Response biomarkers	Related to	Organ/system involvement	Specimen	Value	Availability	Changes or association with response to therapy
IL-1 [66, 100]	immune response	cardiomyopathy	Serum [66]	Increased levels in patients with cardiomyopathy-associated mutations	Clinical work-up	Levels decrease with ERT, in parallel to LVM and interventricular septal thickness reductions
		systemic vasculopathy	Dendritic and macrophage cell culture [100]	Increased levels after exposure to Gb3 plus DGJ	in vitro	
			PBMC [100]	Increased expression levels in classic phenotype	Clinical work-up	No differences in levels between untreated and treated with ERT
IL-6 [25, 66, 78, 100, 101]	immune response	systemic vasculopathy	PBMC [100]	Increased expression levels in classic phenotype	Clinical work-up	No differences in levels between untreated and treated with ERT
			Plasma [25, 101]	Increased levels in ERT treated classic phenotype [25, 101]. Association with urinary Gb3 [101]	Clinical work-up	Not studied
		cardiomyopathy	Serum [66]	Increased levels in patients with cardiomyopathy-associated mutations	Clinical work-up	Levels decrease with ERT, in parallel to LVM and interventricular septal thickness reductions
			Plasma [78]	Increased levels in patients. Association with LVH	Clinical work-up	No differences in levels between untreated and treated with ERT
TNF-α [25, 66, 78, 100, 101]	immune response	systemic vasculopathy	Dendritic and macrophage cell culture [100]	Increased levels after exposure to Gb3 plus DGJ	in vitro	
			PBMC [100]	Increased expression levels in classic phenotype	Clinical work-up	No differences in levels between untreated and treated with ERT
			Plasma [25, 101]	Increased levels in ERT treated classic phenotype [25, 101]. Association with urinary Gb3 [101]	Clinical work-up	Not studied
		cardiomyopathy	Serum [66]	Increased levels in patients with cardiomyopathy-associated mutations	Clinical work-up	Levels decrease with ERT, in parallel to LVM and interventricular septal thickness reductions
			Plasma [78]	Increased levels. Association with LVH	Clinical work-up	Levels are increased in treated respect untreated with ERT
sICAM-1, sVCAM-1 [25, 66, 101]	immune response	systemic vasculopathy	Plasma [25, 101]	Increased levels in ERT treated classic phenotype	Clinical work-up	Not studied
		cardiomyopathy	serum [66]	Increased levels in patients with cardiomyopathy-associated mutations	Clinical work-up	Levels decrease with ERT, in parallel to LVM and interventricular septal thickness reductions

P-selectin [25, 101]	immune response	systemic vasculopathy	Plasma [25, 101]	Increased levels in ERT treated classic phenotype [25,101]. Association with urinary Gb3 [101]	Clinical work-up	Not studied
MCP-1 [66, 104]	immune response	cardiomyopathy	serum [66]	Increased levels in patients with cardiomyopathy-associated mutations	Clinical work-up	Levels decrease with ERT, in parallel to LVM and interventricular septal thickness reductions
		nephropathy	Human podocyte cell culture [104]	Increased expression levels after exposure to Lyso-Gb3	in vitro	
CD74 [42], RANTES [104]	immune response	nephropathy	Human podocyte cell culture	Increased expression levels after exposure to Lyso-Gb3	in vitro	
CD80 [105]	immune response	nephropathy	Human podocyte cell culture	Increased expression levels iafter exposure to Lyso-Gb3	in vitro	
			Urine	Increased urinary levels in patients	Clinical work-up	Not studied
IL-2 [66]	immune response	cardiomyopathy	serum	Increased levels in patients with cardiomyopathy-associated mutations	Clinical work-up	Levels decrease with ERT, in parallel to LVM and interventricular septal thickness reductions
IL-18 [60]	immune response	cardiomyopathy	Cardiomyocytes derived from patients with cardiac variant IVS4 919G>A and cardiomyopathy	Increased expression levels	Clinical work-up	Both enzyme treatment plus IL-18 neutralization slowed the progression of hypertrophy
			Serum and cardiac tissue	Increased levels in patients with LVH compared to controls with VH. Changes in both serum and expression levels associated with progression of hypertrophic cardiomyopathy	Clinical work-up	Levels decrease with ERT, in parallel to LVM and LVMI reductions
MPO [102]	immune response	systemic vasculopathy	Serum	Increased levels. Risk factor for development of vascular event in males	Clinical work-up	No reduction of MPO levels with ERT
TNFR1, TNFR2 [78]	immune response	cardiomyopathy	Plasma	Increased levels. Levels of TNFR2 associated with LVH. Levels of TNFR1, TNFR2 associated with LGE	Clinical work-up	Levels are increased in treated respect untreated with ERT
3-NT [27]	oxidative stress	systemic vasculopathy	Human endothelial cell culture, plasma and aortic tissue homogenate of KO mice	Increased cell culture levels due to loss of a-gal A activity. Increased levels in both plasma and in tissue homogenate	in vitro, animal model	Not studied
			Plasma	Increased levels in ERT untreated classic phenotype	Clinical work-up	Not studied
GSH, GPx activity, TBARS, malondialdehyde, carbonyl groups [101, 103]	oxidative stress	systemic vasculopathy	Plasma, erythrocytes	Altered glutathion metabolism and increased levels [101, 103]. Malondialdehyde and carbonyl gropus associated to urinary Gb3 in ERT treated [101]	Clinical work-up	No marked differences in levels between treated and untreated with ERT [103]
di-Tyr [101]	oxidative stress	systemic vasculopathy	Urine	Increased levels in ERT treated classic phenotype	Clinical work-up	Not studied
NO [103]	oxidative stress	systemic vasculopathy	Urine	Increased levels in ERT untreated classic phenotype	Clinical work-up	No marked differences in levels between treated and untreated with ERT

iNOS, NT [62] 8-OHdG [62, 124]	oxidative stress	cardiomyopathy	Endomyocardial tissue [62]	Increased expression levels in cardiomyocytes of patients with advanced cardiomyopathy. Accumuation of glycosohingolipids	Clinical work-up	Not studied
			Serum [124]	Increased serum levels in patients with cardiomyopathy	Clinical work-up	Levels of 8-OHdG decrease with ERT, in parallel to LVM and LVMI reductions
VEGF, VEGFR2, FGF-2 [54]	angiogenesis	systemic vasculopathy	Bovine aortic endothelial cell culture	Increased expression levels after exposure to Gb3	in vitro	
		nephropathy	Renal tissue homogenate of Fabry mice	Increased expression levels	animal model	Not studied
fibronectin, type IV collagen [42, 55, 104]	ECM turnover	nephropathy	Human podocyte cell culture [42, 104]. Human renal epitelial cell culture [55]	Increased expression levels after exposure to Lyso-Gb3	in vitro	
fragments of collagen alpha-1 (I), (III), (VII) and alpha-3 (V) [63]	ECM turnover	cardiomyopathy	Urine	Correlate with the degree of cardiac fibrosis in non classic phenotype	Clinical work-up	Not studied
PIIINP [63, 124], PIPP, CICT [127]	ECM turnover	cardiomyopathy	Plasma, serum	Increased levels, associated with cardiac fibrosis [127]. PIIINP levels are associated with progression of the degree of fibrosis [63]	Clinical work-up	Not studied
PIPP adjusted for bone turnover [128]	ECM turnover	cardiomyopathy	Serum	Increased levels in patients with cardiomyopathy, including those in early stages. Independent predictor of LVM. Adjusted PIPP:CICT ratio as good diagnostic biomarker to detect LGE fibrosis.	Clinical work-up	No marked differences in levels between treated and untreated with ERT
MMP-2 [78]	ECM turnover	cardiomyopathy	Plasma	Increased levels. Associated with LVH, diastolic dysfunction and LGE	Clinical work-up	Levels are increased in treated respect untreated with ERT
TGF-1 β [42, 54, 55]	fibrosis	systemic vasculopathy	Bovine aortic endothelial cell culture [54]	Increased expression levels after exposure to Gb3	in vitro	
		nephropathy	Renal tissue homogenate of Fabry mice [54]	Increased renal expression levels	animal model	Not studied
			Human podocyte cell culture [42] Human renal epitelial cell culture [55]	Increased (expression) levels after exposure to Lyso-Gb3 or Gb3	in vitro	
E-cadherin, N-cadherin, α-SMA [55]	fibrosis	nephropathy	Human renal epitelial cell culture	Increased (expression) levels of N-cadherin, α-SMA and decreased levels of E-cadherin after exposure to Lyso-Gb3 or Gb3	in vitro	
uromodulin [119, 120, 121]	tubular fibrosis	nephropathy	Urine [119]	Up-regulation at early stages of disease	Clinical work-up	Levels decrease with ERT

			Renal tissue [120]	Abnormal expression profiles in some males, associated with the degree of storage	Clinical work-up	Normalization of abnormal uromodulin expression profiles with ERT
			Urine [121]	Associated with disease progression	Clinical work-up	Not studied
uPAR [106]	degradation of ECM components, regulation of cell morphology, migration and signaling, podocyte injury	nephropathy	Human podocyte cell culture	Increased expression levels after exposure with Lyso-Gb3	in vitro	
			Urine podocytes	Increased expression levels	Clinical work-up	uPAR positive podocytes are reduced in treated respect untreated
Podocin, nephrin, podocalyxin [115, 121]	glomerular permeability, podocyte injury	nephropathy	Urine	Good diagnostic biomarker for males with classic phenotype. Associated with urinary Lyso-Gb3 [115]	Clinical work-up	No marked differences in levels between treated and untreated with ERT
				Increased levels of nephrin and podocalyxin in patients with renal disease, associated with disease progression. Nephrin maybe a sensitive marker of presymptomatic renal disease [121]		Not studied
Cubilin. FGF-23. AMBP [121]	Lipoprotein, vitamin and iron metabolism. Cell differentiation and migration, phosphate homeostasis. Cell adhesion, immune response	nephropathy	Urine	Increased levels in patients with renal disease. Cubulin and AMBP associated with disease progression	Clinical work-up	Not studied
P-p38 [54]	apoptosis	systemic vasculopathy	Bovine aortic endothelial cell culture	Increased expression after exposure to Gb3	in vitro	
		nephropathy	Renal tissue homogenate of Fabry mice	Increased expression levels	animal model	Not studied
caspase-6 and caspase-9 activities [54]	apoptosis	nephropathy	Renal tissue homogenate of Fabry mice	Increased expression levels	animal model	Not studied
S1P [64,122]	cell migration, angiogenesis and vascular maturation	cardiomyopathy	Rat cardiomyocyte cell culture [122]	S1P promotes hypertrophy in cardiomyocytes	In vitro	
			Plasma [64]	Increased levels in males with classic phenotype. Correlate with both carotid artery intima-media thickness and LVM	Clinical work-up	Not studied
hsTnT and ultra-hsTnI [63]	cardiac muscle contraction, cardiac injury	cardiomyopathy	Plasma	Correlate with the degree of cardiac fibrosis in non classic phenotype. Levels of hsTnT are associated with progression of the degree of fibrosis	Clinical work-up	Not studied
BNP, MR-proANP [78]	cardiac remodelling, cardiac injury	cardiomyopathy	Plasma	Increased levels. Associated with diastolic dysfunction and LGE	Clinical work-up	No differences in levels between untreated and treated with ERT
prosaposin, GM2 activator protein [117, 119]	sphingolipid metabolism	nephropathy	Urine	Increased levels at early stages of disease	Clinical work-up	Levels decrease with ERT

prostaglandin H2 D-isomerase [118, 119], complement-c1q tumor necrosis factor-related protein (C1Q/TNF), and Ig kappa chain V-III [118]	immune response, tubular dysfunction	nephropathy	Urine	Increased levels tion pattern of Prostaglandin H2 D-isomerase. Prosaposin H2 D-isomerase	Different glycosylation pattern of Prostaglandin H2 D-isomerase. Prosaposin and Prostaglandin H2 D-isomerase as possible early biomarkers	Clinical work-up	Levels of prostaglandin H2 D-isomerase decrease with ERT [119]

AMBP: Alpha-1-Microglobulin/Bikunin Precursor; α-SMA: α-smooth muscle actin; BNP: Brain natriuretic peptide; CICT: Collagen type I carboxy-terminal telopeptide; DGJ: 1-deoxygalactonojirimycin; ERT: Enzyme replacement therapy; FGF-2: Fibroblast growth factor-2; FGF-23: Fibroblast Growth factor-23; Gb3: Globotriaosylceramide; GSH: Glutathione; GPx: Glutathione peroxidase; hsTn: high sensitive Troponin; IL: Interleukin; iNOS: Inducible nitric oxide synthase; LGE: Late Gadolinium Enhancement; LVH: Left ventricular hypertrophy; LVM: Left ventricular mass; LVMI: Left ventricular mass index; Lyso-Gb3: Globotriaosylsphingosine; MCP-1: Monocyte chemoattractant protein; MMP-2: Matrix metalloproteinase-2; MPO: Myeloperoxidase; NO: Nitric oxide; NT: Nitrotyrosine; 8-OHdG: 8-hydroxydeoxyguanosine; PBMC: peripheral blood mononuclear cells; PIPP: Procollagen type I carboxy-terminal propeptide; PIIINP: Procollagen type III amino-terminal propeptide; proANP: pro Atrial natriuretic peptide; sICAM-1: soluble Intercellular adhesion molecule 1; sVCAM-1: soluble Vascular cell adhesion molecule 1; S1P: Sphingosine-1- phosphate; TBARS: Thiobarbituric acid reactive species; TGFβ-1: Transforming growth factor beta-1; TNF-α: Tumor necrosis factor alpha; TNFR: Tumor necrosis factor receptor; Tyr: Tyrosine; uPAR: Urokinase-type plasminogen activator receptor; VEGF: Vascular endothelial growth factor; VEGFR: Vascular endothelial growth factor receptor.
